# Radiotherapy for early stage favourable breast cancers

**DOI:** 10.1038/sj.bjc.6600004

**Published:** 2002-01-21

**Authors:** T A Buchholz, S E Singletary

**Affiliations:** Department of Radiation Oncology, The University of Texas M.D. Anderson Cancer Center, Houston, TX, USA; Department of Surgical Oncology, The University of Texas M.D. Anderson Cancer Center, Houston, TX, USA

## Abstract

*British Journal of Cancer* (2002) **86**, 309–310. DOI: 10.1038/sj/bjc/6600004 www.bjcancer.com

© 2002 The Cancer Research Campaign

## Sir

We read with great interest the results of a randomized trial investigating the omission of radiation as a component of breast conservation therapy for a favourable subset of women with stage I breast cancer (Holli *et al*., 2001). The data demonstrated that the locoregional recurrence was reduced in the patients randomized to radiotherapy (6.2% (radiation) *vs* 14.1% (no radiation) (*P*=0.029)), but there was no difference in rates of distant metastases or breast cancer deaths. In addition, because the patients who recurred after lumpectomy alone were most often treated with a second lumpectomy and radiation, the final rates of breast conservation were equivalent between the two arms.

A number of points should be considered when interpreting the data of this trial, particularly because these data have the potential of leading to a further decline in the use of radiation following breast conservation surgery. The first point is that the population studied represents a very small subset of women with early stage breast cancer. Despite the study's highly selective eligibility criteria, radiation clearly reduced the risk of local-regional recurrences. These data are consistent with those from previously published prospective trials that also studied highly selected favourable patients and found increased rates of breast recurrences when radiation was omitted (Veronesi *et al*., 1993; Schnitt *et al*., 1996; Liljegren *et al*., 1997; Wollmark *et al*., 2000).

The new twist of this trial is the assertion that breast preservation rates and survival may be equivalent without initial radiation because of the availability of re-excision and radiation as a salvage therapy for those initially treated with surgery alone. In the Holli *et al*. (2001) trial, rates of breast preservation does not appear to have been a predetermined endpoint of the study. The choice of salvage therapy apparently was not determined by the protocol and therefore was likely affected by a number of biases. In addition, the length of follow-up after salvage treatment was not provided and the long-term efficacy of re-excision with or without radiation has not been established. Therefore, we feel that this endpoint should not have been reported.

The goals of all breast cancer therapies are to minimize the risk of breast cancer death and provide the patient with the best quality of life. The authors acknowledge that developing a potentially avoidable local recurrence, even if it is curable with salvage therapy, has a significant negative impact on the quality of life of a patient. A second factor that impacts the quality of life of patients is the final aesthetic outcome of the treatment. The authors stated that radiation can have a negative impact on aesthetics and in their study was avoided in 87.5% of those randomized to the no radiation arm. Aesthetic results are also highly dependent of the extent of surgery. To be eligible for this trial a 1-cm negative microscopic margin was needed. This degree of normal tissue margins requires a considerable volume of breast to be resected and is not necessary for patients receiving radiation. Furthermore, for patients undergoing additional conservative surgery for recurrence, the aesthetic results are likely to be significantly compromised. No reports of aesthetics outcomes are reported for this study.

Finally, a critical question for patients and practitioners to consider is whether increased rates of local recurrences predispose patients to higher rates of distant metastases. This study suggested rates were equivalent, but because of the small number of patients in the study, there were only nine total metastatic events. Furthermore, the authors reported the distribution of these events between the two arms differently in the text of their results section compared to the same data presented in their Figure 2. A large number of patients with a long follow-up period are required to properly study the relationship between locoregional recurrence and distant failure. A recently published meta-analysis that evaluated the outcome of 2091 women treated on breast conservation randomized trials reported that those receiving radiation had a 14% lower relative risk of dying from breast cancer than those patients randomized to not receive radiation (Early Breast Cancer Trialists' Collaborative Group, 2000). This benefit suggests that some local-regional recurrences are associated with new distant metastases and this relationship should be considered when deciding the optimal manner in which early stage breast cancer should be treated.

It is our opinion that sufficient randomized data is available to recommend radiation therapy as a standard after lumpectomy for all women with invasive breast cancers. We feel that omission of radiation should not be offered outside the context of a clinical study.
